# Pathogenesis of fistulating Crohn’s disease: a review

**DOI:** 10.1016/j.jcmgh.2022.09.011

**Published:** 2022-09-29

**Authors:** Colleen Georgette Chantelle McGregor, Ruchi Tandon, Alison Simmons

**Affiliations:** 1 MRC Human Immunology Unit, MRC Weatherall Institute of Molecular Medicine, University of Oxford, Oxford, OX3 9DS; 2 Translational Gastroenterology Unit, John Radcliffe Hospital, Headington, Oxford, OX3 9DU, UK; 3 Pathology, Department of Cellular Pathology, Oxford University Hospitals NHS Foundation Trust, Oxford, OX3 9DU, UK

**Keywords:** Penetrating, Crohn’s-associated fistula, epithelial-to-mesenchymal transition, model systems

## Abstract

Sustained, transmural inflammation of the bowel wall may result in the development of a fistula in Crohn’s disease (CD). Fistula formation is a recognised complication and cause of morbidity, occurring in 40% of patients with CD. Despite advanced treatment, one third of patients experience recurrent fistulae. Development of targeting treatment for fistulae will be dependent on a more in depth understanding of its pathogenesis. Presently, pathogenesis of CD-associated fistulae remains poorly defined, in part due to the lack of accepted *in vitro* tissue models recapitulating the pathogenic cellular lesions linked to fistulae and limited *in vivo* models. This review provides a synthesis of the existing knowledge of the histopathological, immune, cellular, genetic and microbial contributions to the pathogenesis of CD-associated fistulae including the widely accredited contribution of epithelial-to-mesenchymal transition, upregulation of matrix metalloproteinases and overexpression of invasive molecules resulting in tissue remodelling and subsequent fistula formation. We conclude by exploring how we might utilise advancing technologies to verify and broaden our current understanding whilst exploring novel causal pathways to provide further inroads to future therapeutic targets.

## Introduction

Fistulae formation is a recognised complication and cause of morbidity in Crohn’s disease (CD). Chronic, transmural inflammation of the bowel wall in CD, can result in the development of sinus tracts that once penetrating the serosa can result in a fistula (an abnormal communication between two epithelialised surfaces). They can be defined anatomically; those arising internally and those involving the perineum. Internal fistulae may be further classified into those forming an internal communication with another bowel layer (i.e., enteroenteric) or a communication between the intestine and other organs (i.e., enterocutaneous or enterovesical)(1). About 35-40% of patients will develop at least one fistula throughout their disease course(2,3). The majority of fistulae are perianal (54%); however, the less frequent, internal fistula is more difficult to diagnose and treat(2). Ethnic differences are also observed with this phenotype (4,5); South Asian patients with CD are more likely to develop penetrating disease when compared to Caucasians (24.1% *vs* 8.6%)(6).

One third of patients will experience recurring fistulae despite advanced medical therapies and surgical interventions (2). Therefore, novel therapeutic approaches are required to treat fistulating CD, however the prerequisite to this is a better understanding of its pathogenesis.

The pathogenesis of Crohn’s-associated fistulae is poorly defined, in part as accessibility to human tissue from fistulating disease is limiting. In addition, as yet there are no accepted *in vitro* tissue models recapitulating the pathogenic cellular lesions linked to fistulae and limited *in vivo* models to study this phenomenon. Available literature describes the transition of intestinal epithelial cells (IEC) to mesenchymal like cells, upregulation of matrix metalloproteinases (MMPs) and over expression of invasive molecules among the hypothetical pathways involved in the pathogenesis of fistulating CD(7,8). There is little evidence from genetic studies that clearly pinpoints a genetic source for this phenotype, except in the case of early onset monogenic inflammatory bowel disease (IBD)(9,10). The contribution of somatic mutations to this aspect of CD is unknown. Finally, the role of the microbiota or environmental factors to fistulae remains unclear.

Here we present, a synthesis of the current knowledge on the pathogenesis of CD-associated fistulae whilst exploring how we might utilise advancing technologies to expand our understanding of the pathophysiological pathways determining fistulating CD.

## Histopathogenesis

**Figure 1:**
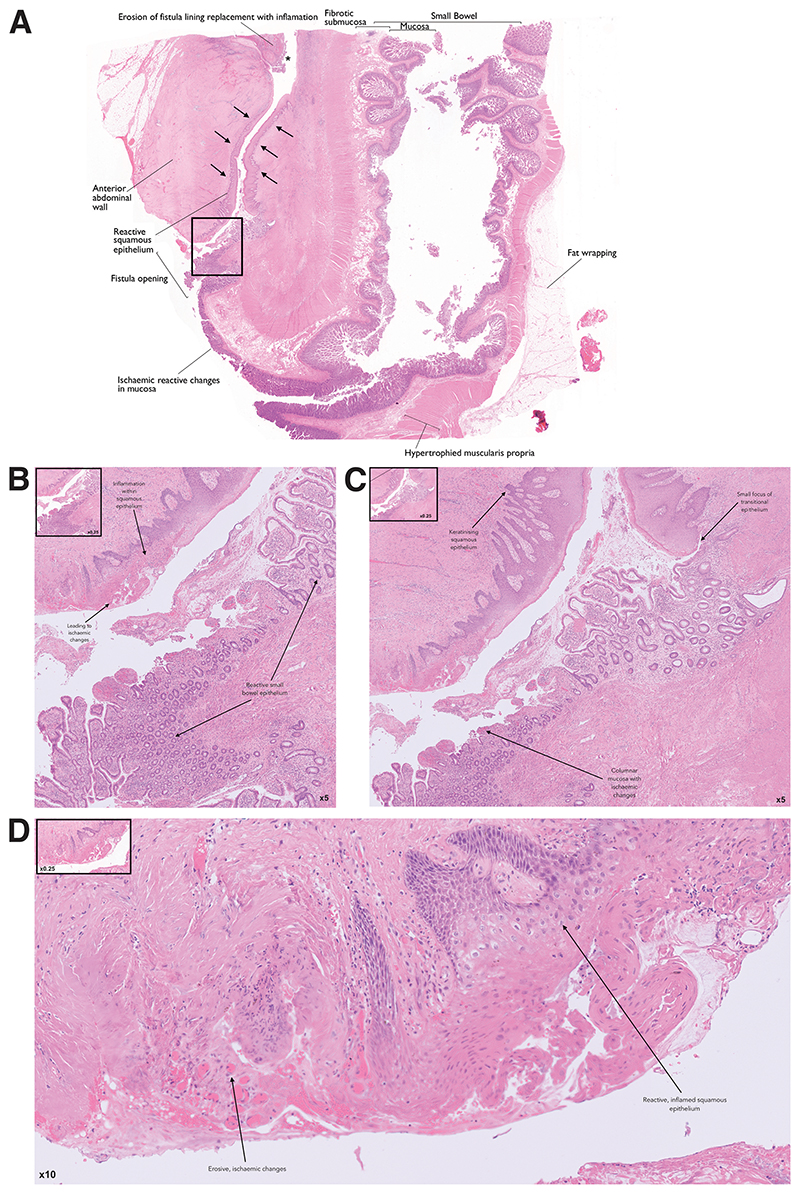
Histopathological image of Crohn’s disease (CD) associated enterocutaneous fistula. **A:** At low magnification, the central fissure of the fistula tract can be identified (arrows) between the anterior abdominal wall and ileum. On the ileal side of the fistula, the mucosa is destroyed with flattened columnar epithelium due to ischaemic / reactive changes in the mucosa. On the cutaneous side of the fistula, features of reactive, narrow squamous epithelium is observed. As observed in the majority of fistulae, erosion of fistula lining is replaced with an inflammatory infiltrate (*). Chronic fibrosis which is a histopathological feature of CD-associated fistulae is characterised here by hypertrophied muscularis propria and fibrotic submucosa within the ileum. Fat wrapping (or creeping fat), which is pathognomonic of ileal CD, is also observed in this specimen. [x0.25 original magnification] **B:** On the ileal side of the fistula, reactive ileal epithelium may be observed. On the cutaneous side, inflammation may be observed within the squamous epithelium leading to ischaemic changes [x5 original magnification] **C:** A small focus of transitional epithelium is observed lining the fistula at x5 magnification. In addition, keratinising squamous epithelium and ischaemic columnar mucosa is seen on the cutaneous side and ileal side of the fistula respectively [x5 original magnification] **D:** Further erosive ischaemic and inflamed changes seen at higher resolution [x10 original magnification] [Haematoxylin-eosin]

Defining CD-associated fistulae histopathologically is vital to understanding its pathogenesis. A study examined several fistula specimens, both CD-associated and controls, histopathologically (11). All fistulae, independent of underlying cause, were characterised by a central fissure penetrating the lamina propria and muscularis mucosae through to underlying tissue layers. Universally, fistulae were surrounded by granulation tissue with histiocytes and a dense network of capillaries. The luminal contents may include debris, erythrocytes or non-specific acute or chronic inflammatory cells (11). Granulomas may be present in CD-associated fistulae however, they are not disease specific; for instance, granulomas are absent in most CD-associated perianal fistulae (12). Chronic fibrosis may also be a histopathological feature of CD-associated fistulae (Figure 1).

Epithelium lining fistulae is typically flattened in the small or large intestine, or consists of narrow squamous cells in perianal or cutaneous fistulae (Figure 1). Not all fistulae have an epithelial lining however; two thirds are non-epithelialised. Non-epithelialised CD-associated fistulae are instead lined by mesenchymal-like cells, termed ‘transitional cells’, with retained gap junctions to each other. This thin layer of transitional cells (TC) represents epithelial cells thought to have undergone transformation to mesenchymal-like cells. In some sections, a new basement membrane form with these TC connected by fibronexus. In other sections, TC may appear more disordered with no visible gap junctions and fragmented basement membranes. These features appear unique to CD-associated fistulae when compared to controls (11).

The majority of fistula specimens demonstrate inflammation, with severe acute inflammation in at least 56% (11). Inflammatory cell populations have been studied immunohistochemically; in patients with CD-associated fistulae, the inner fistula wall is densely infiltrated with CD45RO^+^ T cells and a small band of CD68^+^ macrophages. The outer fistula wall is marked by CD20^+^ B cells. This is in contrast to non-CD-associated fistulae, whereby CD68^+^ macrophages are seen throughout the whole fistula wall with CD45RO^+^ T cells occupying the outer two thirds of the fistula wall. There are also considerably fewer B cells. The distribution of immune cells is independent of the fistulae location or the depth of penetration (11).

Maggi and colleagues performed phenotypic and functional analysis of T-cells recovered from CD perianal fistulae tissue and circulating T-cells from peripheral blood (13); CD4^+^CD161^+^ T-cells with Th17, Th1, Th17/1 phenotype significantly infiltrate fistula tissue compared to peripheral blood. Locally administered anti-TNF (Adalimumab) resulted in a resolving fistula clinically and a significant reduction in the frequency of CD161^+^ T-cells within fistula tissue.

A further study identified significant differences in T-cell subsets in fistulating CD when compared to healthy and non-fistulating CD, specifically the authors detected an increase of CD3^+^CD8^-^ T-cells and a decrease in CD3^+^CD8^+^ T-cells in peripheral blood. Both T-cell subsets secreted high amounts of TNF-*α* and IL-13 in co-culture experiments, both of which are recognised as key cytokines in the pathogenesis of fistulating CD (see below)(14).

## Immune and cellular biology

### The epithelial-to-mesenchymal transition theory

Epithelial-to-mesenchymal transition (EMT) is a process by which an epithelial cell differentiates into a mesenchymal-like cell, acquiring its features and properties. Epithelial cells lose their defining characteristics such as polarity and adhesiveness and adopt a mesenchymal phenotype such as reduced cell-to-cell adhesion and enhanced migratory potential (15,16).

In health, EMT plays a critical role in embryogenesis and organ development (17,18). EMT is associated with the ability to migrate and penetrate into adjacent tissue layers. In disease, EMT may be seen contributing to cancer and fibrosis (19–21). EMT plays a pivotal role in tissue remodelling, occurring in response to tissue damage where there is a need to recruit mesenchymal cells from epithelial cells. Whilst the epithelial cells acquire the ability to migrate to sites of injury through EMT, dual effects of excess extracellular matrix (ECM) deposition secreted by mesenchymal like cells can result in tissue fibrosis (22). The shift from epithelial to mesenchymal cell is orchestrated by a wide range of cellular changes such as expression of transcription factors, cytokines and regulatory proteins (9).

**Figure 2:**
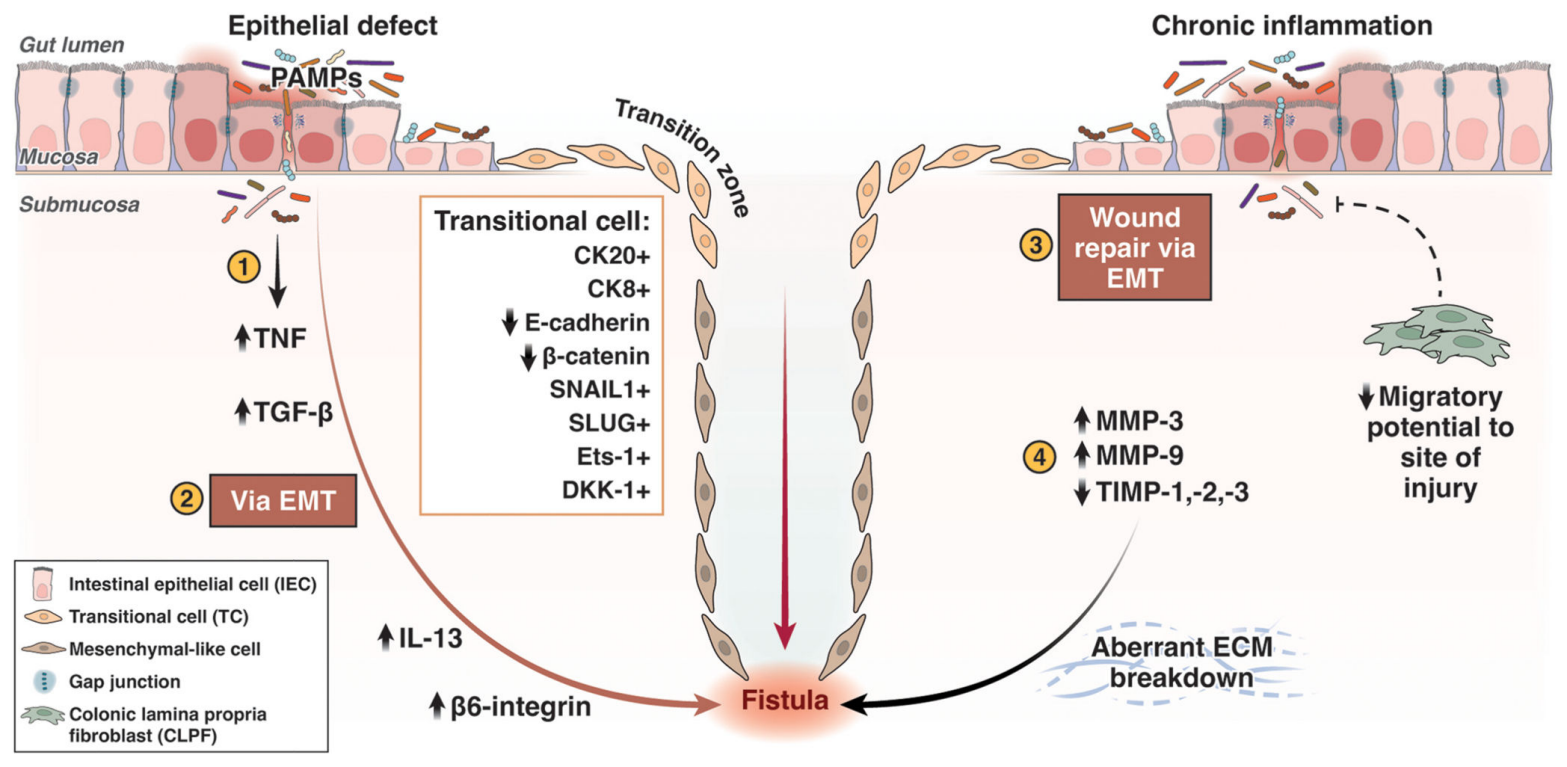
Schematic of the pathogenesis of CD-associated fístula formation (1) IEC undergo EMT converting to TC in response to a defect in the epithelial barrier; resultant infiltration of pathogen associated molecular patterns enter the gut mucosa which elicits an immune response. (2) An upregulation of TNF, a potent inducer of TGF-β, occurs which stimulates a cascade of pro-inflammatory cytokines (IL-13) and cell invasive molecules (β6-integrin) resulting in TC adopting features of an invasive mesenchymal-like cell. TC preserve their epithelial origins (CK20/8+) however down-regulate expression of epithelial cell adhesion molecules (E-cadherin, β-catenin) and highly express EMT-inducing transcription factors (SNAIL1, SLUG, Ets-1) and cell migratory molecule, DKK-1. In addition, TCs may appear more disordered with a loss of gap junctions and fragmented basement membranes. (3) Chronic inflammation results in reduced epithelial repair and reduced migratory capabilities of CLPF which contribute to poor wound healing in fistulating CD; IEC compensate by undergoing EMT in an attempt to restore the epithelial barrier. (4) Matrix metalloproteinases (MMP-3, MMP-9) are highly expressed and unopposed (reduced TIMP-1, -2, -3) in fistulating CD tissue resulting in aberrant breakdown of the extracellular matrix and tissue remodelling. These pathways consequently contribute to fistula formation. [Figure created with BioRender.com and adapted from Siegmund et al (12); Abbreviations: CD, Crohn’s disease; CK, Cytokeratins; DKK-1, Dickkopf-homolog 1; ECM, extracellular matrix; EMT, epithelial–to-mesenchymal transition; ETS-1, E-twenty-six; IEC, intestinal epithelial cell; IL-13, interleukin 13; MMP: Matrix metalloproteinase; PAMPs, pathogen-associated molecular patterns; TC, transitional cell; TGF, tissue growth factor; TIMP, tissue inhibitor of MMP; TNF, tumour necrosis factor]

Much data provides supporting evidence for the theory that EMT drives CD-associated fistula formation (11,23,24). Chronic inflammation results in both reduced epithelial repair and reduced migratory capabilities of colonic lamina propria fibroblasts (CLPF) which contribute to poor wound healing in fistulating CD (25,26). Within a fistula, IEC compensate for defective CLPF by converting to TC in an attempt to restore the intestinal epithelial barrier (11)(27). TC form a thin monolayer, lining the fistula. The region where IEC transform into TC is referred to as the ‘transitional zone’. TC appear unique to CD-associated fistulae. Present in ‘non-epithelialised’ areas of fistula, TC express epithelial markers cytokeratin 8 and 20, reflecting their epithelial origins. Expression of epithelial adhesion markers, E-cadherin and *β*-catenin is downregulated however (23). Both proteins facilitate cell-to-cell adherence, therefore their downregulation allows the cell to adopt migratory properties – a defining role of EMT (Figure 2).

Previous studies defining CD-associated fistulae immunohistochemically, have demonstrated the presence of potent inducers and markers of EMT (23,28). *β*6-integrin is overexpressed in the transitional zone of TC in CD-associated fistulae (23); this overexpression of *β*6-integrin correlates with increased cell invasiveness.

Severe intestinal inflammation results in the secretion of cytokines TNF-*α*, IL-13 and TGF-*β*. TGF-*β* is the most potent inducer of EMT. It results in the induction of transcription factors in IEC associated with EMT such as Ets-1, SNAIL1 and SLUG (SNAIL2). The SNAIL family transcription factors strongly repress E-cadherin. SNAIL1 has been shown to be highly expressed in the nuclei of TC lining CD-associated fistulae; SLUG (SNAIL2) in contrast is mainly limited to cells around the fistula tract (29). In addition, Ets-1 is highly expressed in CD-associated fistulae (24,30). Ets-1 mediates the activation of *β*6-integrin and therefore enhances cell invasion during EMT. Upregulation of TGF*β*-1 and TGF*β*-2 expression is also seen in TC lining CD-associated fistula tracts when compared to normal IECs (23). Its upregulation is also linked to *β*6-integrin expression (23,31).

In summary, strong features to suggest EMT in CD-associated fistulae include: Reduced E-cadherin and *β*-catenin expression, upregulation of TGF-*β*, induction of EMT transcription factors (SNAIL1, SLUG & Ets-1) and *β*6-integnn overexpression in TC - all resulting in enhanced migratory potential and increased cell invasiveness. These features appear to be consistent irrespective of fistula location (16) (Figure 2).

Dickkopf-related protein 1 (DKK-1) is an important factor in the regulation of cell migration through its ability to block IEC migration. Expression of DKK-1 is strongly upregulated in the TC lining CD-associated fistula tracts with weak expression in healthy controls (32,33). New findings propose its function as a Wnt inhibitor may regulate TGF-*β*-stimulated IL-13 secretion and therefore EMT in IEC (33).

More recently, Ortiz-Masiá and colleagues demonstrated that the levels of metabolite succinate and expression of its receptor SUCNR1 were significantly increased in fistulating CD tissue. SUCNR1 increases expression of Wnt ligands, activates Wnt signalling pathways which in turn induces EMT in IEC (28). Previous work from the same authors, show that increased EMT induction in fistulating CD tissue is driven by an increased interaction between Wnt ligand, WNT2b and receptor FDZ4 when compared to controls (32).

### Cytokine profile

Published data has sought to define the cytokine profile of CD-associated fistula tracts and therefore glean its immunopathogenesis. Tumour necrosis factor (TNF) induces EMT in IEC and can induce expression of TGF (30,34,35). TNF and its receptor, TNF Receptor 1 (TNF-R1) are strongly expressed in TCs lining fistulas along with IEC of adjacent crypts in patients with CD (29). In addition, in both IEC and CLPF, TNF induces *β*6-integrin and Ets-1 transcription factor, both key mediators of EMT (30). Furthermore, serum TNF-*α* levels significantly correlate with the presence of active CD perianal fistulae (36).

Similar to TNF, IL-13 and its receptor, IL-13R1 are heavily expressed in TC lining fistula tracts and adjacent crypts. This appears unique to CD-associated fistulae, as IL-13 expression is notably absent in healthy intestine, UC and non-fistulating CD, regardless of inflammation. IL-13 upregulates TNF-*α*, IL-12 and IL-6 in fistulating tissue (24). Functionally, IL-13 promotes genes involved with cell invasion (*β*6-integrin) and EMT (SLUG) in IEC and in *in vitro* models of EMT.

A study examining cytokine concentrations in fistula tracts of idiopathic versus perianal CD fistulae demonstrated significantly higher IL-12 concentrations and a lower IL-1RA/IL-1β ratio in the CD group (37).

TGF-*β*; the key mediator of EMT also localises to the TC lining fistula tracts and induces SNAIL1 and IL-13 in *in vitro* models. Primary human CLPF derived from patients with fistulating CD demonstrated altered function when treated with TGF-*β*. TGF-*β* and IL-13 are thought to display synergism in the pathogenesis of fistulae (24).

The abundance of these cytokines in the lining of fistula tracts, adjacent tissue and peripheral blood implies their involvement in fistulating CD pathogenesis. Furthermore, the significance of the role of cytokines is further substantiated by the clinical efficacy of anti-cytokine biological agents such as Infliximab (anti-TNF-*α*) in the treatment of fistulating CD(38).

### MMPs & TIMPs

MMPs have tissue degrading and remodelling properties. Aberrant ECM breakdown secondary to MMP activity can lead to cancer or IBD. Increased MMP activity is associated with immune-mediated tissue injury and is found in CD (39). Murine models of DSS-colitis demonstrate the significance of MMP, with selected deletion of MMP-9 conferring a protective effect (40–43). Tissue inhibitors of MMP (TIMPs) are the natural inhibitors of MMP, secreted by MMP producing cells (44). Kirkegaard and colleagues identified strong expression of MMP-3 in CD-associated fistula tissue when compared to controls, irrespective of inflammatory state. MMP-3 were largely localised to mononuclear cells and fibroblasts, with MMP-9 predominantly in granulocytes and only in fistulae with active inflammation (45). MMP-3 and MMP-9 have also been identified in idiopathic fistulae. MMP-13 protein expression is also detectable in CD-associated fistulae but almost absent in non-fistula CD tissue. Protein levels of inhibitory molecules TIMP-1, TIMP-2 and TIMP-3 are correspondingly low in CD-associated fistulae tissue (45). This provides evidence for the basis of MMPs as mediators in the pathogenesis of CD-fistulae through aberrant ECM degradation.

## Genetic contribution

The interactions between genetics and environment are well described in CD. Extensive genomewide association studies have identified approximately 240 genes associated with pathogenesis or risk of disease (46–48) Genetic contributions per fistulating phenotype have also been described (49–51). Nucleotide oligomerization domain 2 (*NOD2*) remains the strongest genetic predictor of CD susceptibility and phenotype including fistulating disease (51–53). Henckaerts at al demonstrated the following as independently associated with non-perianal CD fistulating phenotype; the presence of a T-allele at rs12704036 (OR 1.74), followed by the presence of any *NOD2* variant (OR 1.47) and *IRGM* rs4958847 (OR 9.22)(49).

The presence of a C allele at *CDKALI* rs6908425 and the absence of any *NOD2* variant was associated with perianal fistulating phenotypes (p=0.008 and p=0.002 respectively)(49). Conversely, in latter work, *NOD2* mutation, rs72796353 was found to be significantly associated with perianal fistula development (OR 5.27, p=2.78 x 10^-7^) when compared to *NOD2* wild-type carriers (54).

In addition to *NOD2* polymorphisms, the risk of developing internal penetrating CD is significantly associated with the carriage of a variant allele of *PRDM1* rs7746082, LOC441108 and *IL23R*. Specifically, the carriage of *ATG16L1* and *PRDM1* are independently associated with an earlier onset of internal penetrating disease in contrast to *IL23R* which is associated with a later onset(51). OCTN variants have also been demonstrated in association with penetrating disease behaviour in both Korean (OR 4.23)(55) and Belgian (OR 1.47)(56) populations. The gene *PUS10* confers a protective effect against perianal fistulising disease (51).

More recently, epigenetic analysis defined an intestinal mucosa-derived DNA methylation signature in fistulating CD mucosal lesions; differential DNA methylation sites were enriched in the upregulation of apoptotic processes and IL-8 production when compared with normal intestinal mucosal tissue (57).

It is noteworthy that genetic contributions to the CD fistulating phenotype differ in perianal and internal fistulae. However, universally the risk alleles associated with fistulating CD encode for proteins involved with regulation of ileal microbiota, adaptive immunity or maintaining the integrity of the intestinal epithelial barrier.

## Microbial contribution

The host-microbe interaction in CD is well studied (48,58), less well characterised is the role of the intestinal microbiome in CD-associated fistula development and persistence. There is a rationale to suggest a bacterial contribution to the aetiology of fistulae given the efficacious role of antibiotics in its management, namely in perianal fistulae. A limited number of studies have explored the microbial composition of CD-associated fistula tracts to characterise potential causative organisms. In a cohort of thirteen patients with CD-perianal fistulas, West and colleagues demonstrated that perianal fistulae are predominantly colonized with gram-positive microorganisms(59). Another study showed that gram-positive bacterium *Corynebacterium* and gram-negative *Achromobacter* were significantly more abundant in perianal fistula tract samples relative to matched stool samples via 16S rRNA gene profiling (60). In contrast, a study examining idiopathic and Crohn’s anal fistula tracts did not isolate any mucosa-associated bacteria despite the presence of mucosal inflammation. Whilst the authors acknowledged potential for sampling error, they also suggest that established fistula tracts devoid of bacterial colonisation may imply that bacteria are unimportant for fistula persistence(61).

Bacterial cell wall muramyl dipeptide (MDP), for which *NOD2* is a receptor for, induces expression of molecules relevant to EMT (TNF-α, TGF-β, SNAIL1, IL-13 and Ets-1) within IEC and lamina propria fibroblasts from fistulae (30). Furthermore, it has been reported that certain enteric pathogens, such as *Citrobacter rodentium* and *E*. *coli*, can trigger the onset of EMT through activated signalling pathways, implying that luminal bacteria may play a role in fistula development through the theorised EMT pathway (62,63).

Emerging data is now defining the role of the mycobiome in CD; Jain et al demonstrate that fungus, *Debaryomyces hansenii* is enriched in inflamed intestinal CD tissue and can cause dysregulated mucosal healing (64). Such alterations in tissue repair capacity may contribute to the pathogenesis of CD-associated fistula. The natural competition between fungi and bacteria within the gut is disturbed with the use of antibiotics. It is feasible that with the elimination of certain bacteria, impaired mucosal healing may occur due to the unopposed action of and relative enrichment of fungi within the gut and may give explanation to the persistence of perianal fistulae, in some cases, despite antibiotics.

With advancing technologies, future work needs to both refine and broaden our understanding of the potential role of the microbiome, including the mycobiome, in CD-associated fistulae.

## Future avenues

Historically, perianal CD-associated fistulae have been better phenotyped principally due to easier access to tissue. Direction of future work must first begin with accessing lesional tissue from CD-associated intestinal fistulae. Using novel methods, researchers must first phenotype human lesional tissue and define the microenvironment of CD-associated fistulae. The understanding of which will inform the conditions or pathways in which *in vivo* models might be developed and explored. Secondly, leveraging existing knowledge of the phenotypic and molecular characteristics of idiopathic fistulae (45,65,66) will be a first flourish in understanding the distinct and shared pathways of CD-associated fistula pathogenesis.

### In vitro models

To date, the use of *in vitro* models (e.g., intestinal epithelial cell lines) and descriptive histopathological and immunohistochemical data has been the main source of knowledge of fistulating CD pathology (14,23,32). Meier and colleagues successfully developed a 3D matrix model to study the behaviour of CD-CLPFs isolated from CD-associated fistulae and strictures. Patient derived CLPFs were cultured and seeded into a 3D matrix, the CLPF layer underwent laser wounding with subsequent study of cell migration assays. Fistula-associated CLPFs were found to have significantly reduced migratory potential (27).

Remarkable advances in the development of intestinal epithelial organoids (IEOs) now offer a more accurate representation of intestinal epithelial structure and function in health and disease (67,68). Organoid based models have also provided further evidence for EMT in intestinal diseases such as CD (69–73). Pivotal work from Hahn et al demonstrated the ability of TNF-*α* and TGF-β to induce EMT in IEOs; mesenchymal phenotypic changes were observed in the TGF-β1 stimulated IEOs. Proposing a novel model for studying EMT in intestinal fibrosis (69).

Moreover, sequencing technology advances permit identification of somatic mutational burden within epithelia of patient-derived organoid cultures (74). Future work should focus on developing a reproducible intestinal fistula-derived organoid model including both epithelial and stromal compartments. By manipulating the inflammatory microenvironment in such organoids, future work will clarify the possible contribution of EMT in CD-associated fistulae formation and explore whether said process may be stopped or indeed reversed.

Finally, recent advances in tissue engineering and organoid methodologies might serve as an exciting prospect to model CD-associated fistulae *in vitro*. Meran et al successfully generated decellularized intestinal tissue scaffolds with resected human intestines seeded with patient-derived intestinal organoids (75). Leveraging such methods could be the key next step in model development.

### In vivo models

However, a major limitation in the field of fistulating CD research is the lack of a suitably sufficient and reproducible animal model. Only a few animal models have shown promise in their ability to develop intestinal fistulae. Firstly, Cominelli’s group established a sub strain of the SAMP1/Yit mouse associated with spontaneous perianal fistula formation (76). The SAMP1/YitFc mouse, generated by sibling mating over twenty generations exhibited fistulating perianal disease in 4.8% of the colony. The disease was characterised by mucosal ulceration of the anal canal, fissures, perirectal abscesses and anocutaneous fistulae. Multiple fistulae were not observed however. Whilst this model is arguably the closest animal model of CD with its co-existing terminal ileitis; the low incidence of perianal fistula limits its widespread use as a fistulating model.

Enterocutaneous fistula formation in a human gut xenograft is another novel model whereby Bruckner and colleagues(77), transplanted human foetal gut segments subcutaneously into mature SCID (C.B-17/IcrHsd-Prkdcscid) mice. Approximately 17% developed spontaneous enterocutaneous fistulae, notably in those with a lack of IL-10 response. Histopathological findings showed remarkable similarities with CD-associated fistulae including evidence for EMT immunohistochemically(78). Whilst promising, this model requires further validation and replication. Several mouse models of non-intestinal fistula exist including trachea-oesophageal (79), and vascular fistulae (80), which may also serve to inform development of future CD models.

## Conclusions

Fistulating CD is a complex clinical entity with significant morbidity and mortality to the patient and economic burden to healthcare. Development of targeting treatment for fistulae will be dependent on a more in depth understanding of pathogenesis. The most accredited theory for its pathogenesis remains EMT with several inducers of EMT identified in CD-associated fistula tissue and validated with *in vitro* models. To date, data on EMT has largely been captured by RT-PCR, co-culturing and immunohistochemistry techniques. Multimodal single cell analysis will enable definition of cell circuits driving this phenomenon. Understanding the contribution of the tissue niche around fistulating tissue and epithelial-stromal crosstalk unique to fistulating CD will be key to advancing the field.

While aberrant epithelial-stromal crosstalk is important, the wider cellular milieu also plays a role. Recent data on the significance of the mesentery in the pathogenesis CD is of interest (81); the mesentery, including lymphatics, adipose tissue and nerves, host metabolic and immune properties (82) and may indeed have a role in fistulating CD. The exact mechanisms of which are unknown and warrant further study.

The differential risk of fistulating CD by ethnicity is considered with interest; the contribution of environment and genetics by ethnicity is unclear. Whilst a single genetic source does not pinpoint the fistulating phenotype, it is noteworthy that much of the literature on genetic contributions in IBD is derived from Caucasian populations. Similarly, the role of environmental factors in fistulae formation is underdeveloped.

In summary, our current understanding suggests that the pathogenesis of fistulating CD is multifactorial – a combination of EMT and overexpression of MMPs in response to an epithelial defect mediated by the upregulation of pro-inflammatory cytokines, genetic susceptibility, epigenetic changes and possible influence of the intestinal microbiota. The development of robust *in vivo* model systems of fistulating CD is required to verify and broaden our current understanding whilst exploring novel causal pathways to provide further inroads to the pathogenesis of fistulating CD.

## Synopsis

Fistulating Crohn’s is a complex clinical entity; its pathogenesis appears multifactorial – a combination of EMT, upregulated matrix metalloproteinases and pro-inflammatory cytokines together with genetic and microbial contributions. Development of *in vivo* models are required to verify and broaden current understanding.

## Abbreviations

CLPF: Colonic lamina propria fibroblasts
CD: Crohn’s disease
DKK-1: Dickkopf-related protein 1
ECM: extracellular matrix
EMT: epithelial-to-mesenchymal transition
Ets: E-twenty-six
IBD: Inflammatory bowel disease
IEC: Intestinal epithelial cells
IEOs: Intestinal epithelial organoids
MMPs: Matrix metalloproteinases
MDP: Muramyl dipeptide
*NOD2*: Nucleotide oligomerization domain 2
PAMPs: Pathogen-associated molecular patterns
TC: Transitional cells
TIMPs: Tissue inhibitors of MMP
TNF: Tumour necrosis factor
TNF-R1: TNF Receptor 1
